# Influence of acetylene on methane–air explosion characteristics in a confined chamber

**DOI:** 10.1038/s41598-021-93466-4

**Published:** 2021-07-06

**Authors:** Jinzhang Jia, Jinchao Zhu, Wenxing Niu, Jing Zhang

**Affiliations:** 1 College of Safety Science and Engineering, Liaoning Technical University, Fuxin, 123000 Liaoning China; 2 Key Laboratory of Mine Thermo-Motive Disaster and Prevention, Ministry of Education, Huludao, 125105 Liaoning China; 3 Tianjin Jinpri Environmental Technology Co., Ltd., Tianjin, 300000 China

**Keywords:** Chemistry, Energy science and technology, Engineering

## Abstract

To study the impact of acetylene on methane explosions, the safe operation of coal mines should be ensured. In this paper, a 20 L spherical tank was used to study the explosive characteristics of acetylene–methane–air mixture. In addition, the GRI-Mech3.0 mechanism was used to study the chemical kinetic mechanism for the mixed gas, and the effect of adding acetylene on the sensitivity of methane and the yield of free radicals was analysed. The results show that acetylene can expand the scope for methane explosion, lower the lower explosion limit, and increase the risk of explosion. Acetylene increases the maximum explosion pressure, laminar combustion rate and maximum pressure rise rate for the methane–air mixture while shortening the combustion time. Three combustion modes for the acetylene–methane–air mixture were determined: methane-dominated, transitional and acetylene-dominated combustion modes. Chemical kinetic analysis for the mixed gas shows that as the volume fraction of acetylene increases, the generation rate for key free radicals (H*, O* and OH*) gradually increases, thereby increasing the intensity of the explosive reaction. The results from this research will help formulate measures to prevent coal mine explosion accidents.

## Introduction

Methane is a widely used energy source and the main component of natural gas and coal mine gas. In coal mining, affected by geological conditions, many gases may accumulate^1^. These substances mixed with methane can lead to an explosion hazard and pose a safety hazard. Therefore, in-depth studies of mixed gas explosions should be conducted.

In recent years, scholars have studied the explosive characteristics of mixed combustible gases through experiments. This research has mainly focused on methane and other gases (mainly CO, H_2_, alkane gas, olefin gas, and other gases). Experiments have obtained the explosion limit, explosion pressure parameters, flame propagation characteristics, and laminar burning velocity of mixed gas^2–8^. The results have shown that combustible gases can result in significant changes in the explosion characteristics of methane. This may be related to the fact that enrichment of methane with other gases aggravates the severity of an explosion and should be related to the strong nonlinear effects of laminar and turbulent flame propagation^9,10^. In addition, this phenomenon can also occur in a mixed explosion of coal dust and methane^11,12^. Researchers have also conducted inerting experiments for mixed gas explosions and studied the flame structure, explosion characteristics, and laminar burning velocity of mixed gas explosions. The result is that N_2_, CO_2_, NaCO_3_, water mist, argon, and other substances can inhibit gas explosions and reduce the harm caused by explosions^13–17^. The above research provides data support for preventing gas explosions and reducing the risk of explosions.

With gradual progress in research, scholars have applied chemical mechanism analysis and mathematical models based on quantum chemistry to analyse gas explosion mechanisms. Nie et al.^18^ obtained the main factors affecting the chemical kinetics of methane explosions through numerical simulation. Liang and Zeng^19^ found that water can prolong a gas explosion and reduce the concentrations of H*, O*, and OH*. Raul et al.^20^ studied the internal mechanism of methane oxidation under explosive conditions and found that OH* radicals are the critical factor leading to methane explosion. Su et al.^21^ and Luo et al.^22,23^ used a monochromator and oscilloscope to obtain flame spectrum data and discovered the key process for methane explosion.

Most of the above studies have been concerned with the influence of olefins, alkanes, inert gases, solid particles, etc. on the explosion of methane. There are relatively few explosion safety data, such as the explosion limit for the mixed explosion of alkyne and methane, the explosion pressure parameters and the laminar combustion velocity. According to the experimental results obtained for other mixed gases mixed with methane, the explosion hazard for methane-acetylene mixtures is very high, and the explosion phenomenon will release much energy, resulting in serious consequences. Simultaneously, spontaneous combustion of coal also produces acetylene^24^. This may lead to a mixed explosion of methane-acetylene, thereby giving rise to potential safety hazards for safe coal mine production. Therefore, it is essential to study the explosion of methane-acetylene-air mixtures.

This paper studies the explosion characteristics of acetylene–methane–air mixtures and the change in explosion limit through experiments, analyses the chemical reaction mechanism of methane-acetylene-air explosions, and obtains the change in methane sensitivity and the production of free radicals in explosions.

## Experimental equipment and research method

### Experimental method

#### Equipment

**Figure 1:**
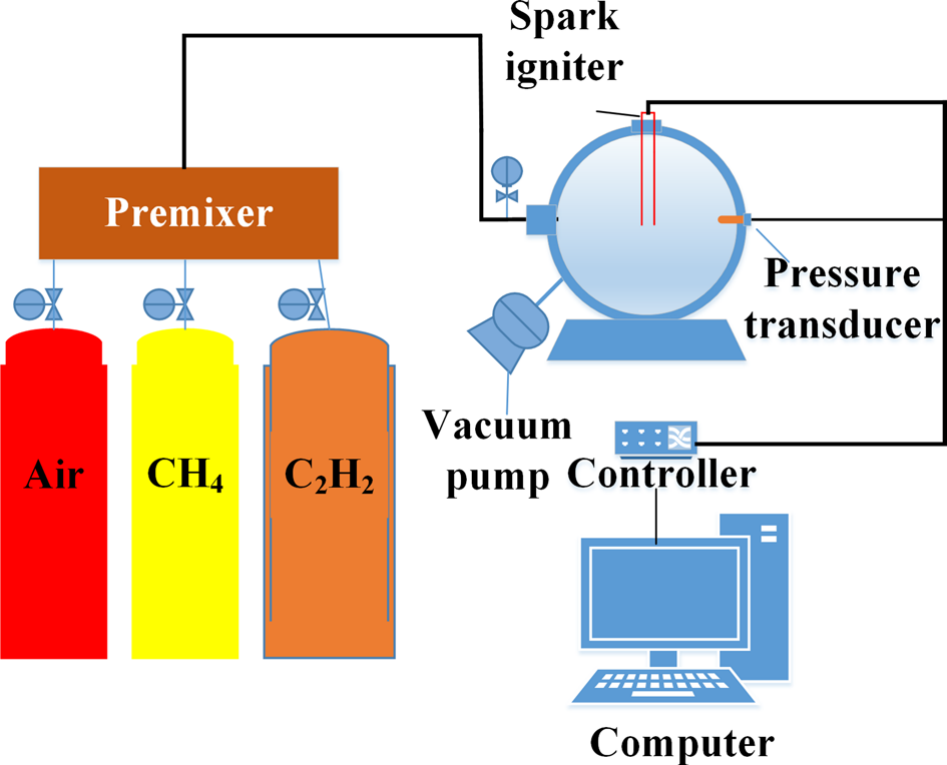
Experimental equipment.

In past research, scholars have studied the effects of containers of various sizes and shapes on the explosion characteristics and found that the results have reasonable minor deviations, so this paper selected a 20 L spherical container for experiments^25^. Figure 1 shows the specific experimental equipment used, including a 20 L spherical explosion tank, a data acquisition instrument, electric spark generator, vacuum pump, high-pressure gas cylinder, and other parts. The accuracy of the mixed gas system is 0.1%. The electric spark generator is arranged on the upper part of the tank and consists of a probe and a high-voltage pulse generator. The ignition position is at the center of the spherical explosive tank, and the ignition energy is 1 J. The sampling frequency of the piezoelectric sensor is 5 kHz, with a response time of 0.1 ms and a relative error of 0.2%. The dynamic pressure from ignition to 2000 ms can be collected through a data acquisition instrument and computer. The environmental conditions for the experiment were as follows: temperature of 298 K, air pressure of 0.1 MPa, and ambient humidity of 48–59%.

#### Procedure

The experiment in this paper was divided into two parts. The first part investigated the influence of a low concentration of acetylene on the explosion limit of methane, using the volume fraction as the unit; the second part of the experiment investigated the acetylene–methane–air mixture explosion characteristics in terms of rich combustion, stoichiometric ratio, and oxygen enrichment, using the equivalent ratio as the unit.

In the first part of the experiment, assuming that the lower explosive limit of pure methane tested in the experiment was 5.2%, the amount of acetylene gas added was 0%, 0.4%, 0.8%, 1.2%, 1.6%, and 2.0% of the volume fraction (the addition amount is relative to the methane–air mixed gas). The volume fraction of methane decreased from 5.2% until the critical value of explosive and non-explosive mixtures is found, with a decrease of 0.1% for each step. The lower explosive limit for the mixed gas was the average of the two, and the upper explosive limit for the mixed gas was measured in the same way.

The second part of the experiment used four concentration equivalent ratios (0.8, 1, 1.2, 1.4) and six acetylene volume ratios (0%, 20%, 40%, 60%, 80%, 100%) to explode the methane-acetylene-air mixture in the test. The mixture ratio was calculated using Formulas (1) and (2):

1 $$\Phi {\text{ = (}}F{\text{/}}A{\text{)/(}}F{\text{/}}A{\text{)}}_{{{\text{stioch}}}}$$

2 $$X{\text{ = }}{{V_{{{\text{acetylene}}}} } \mathord{\left/ {\vphantom {{V_{{{\text{acetylene}}}} } {(V_{{{\text{acetylene}}}} + V_{{{\text{methane}}}} )}}} \right. \kern-\nulldelimiterspace} {(V_{{{\text{acetylene}}}} + V_{{{\text{methane}}}} )}}$$

where *V*acetylene and *V*methane are the volume fractions of ethylene and methane, respectively. (*F*/*A*) is the actual ratio of fuel to oxygen in the test sample, and (*F*/*A*) Stioch is the stoichiometric fuel/oxygen ratio.

#### Data specification

This article judges the explosion situation by observing the pressure change. According to the American Society for Testing and Materials (ASTM) standards, an explosion occurs when the pressure increases by 7% or more^26,27^. Each experiment was repeated three times to ensuring accuracy for the experimental results and controllable errors. The experimental data for the pressure parameters were expressed by (mean + variance).

**Figure 2:**
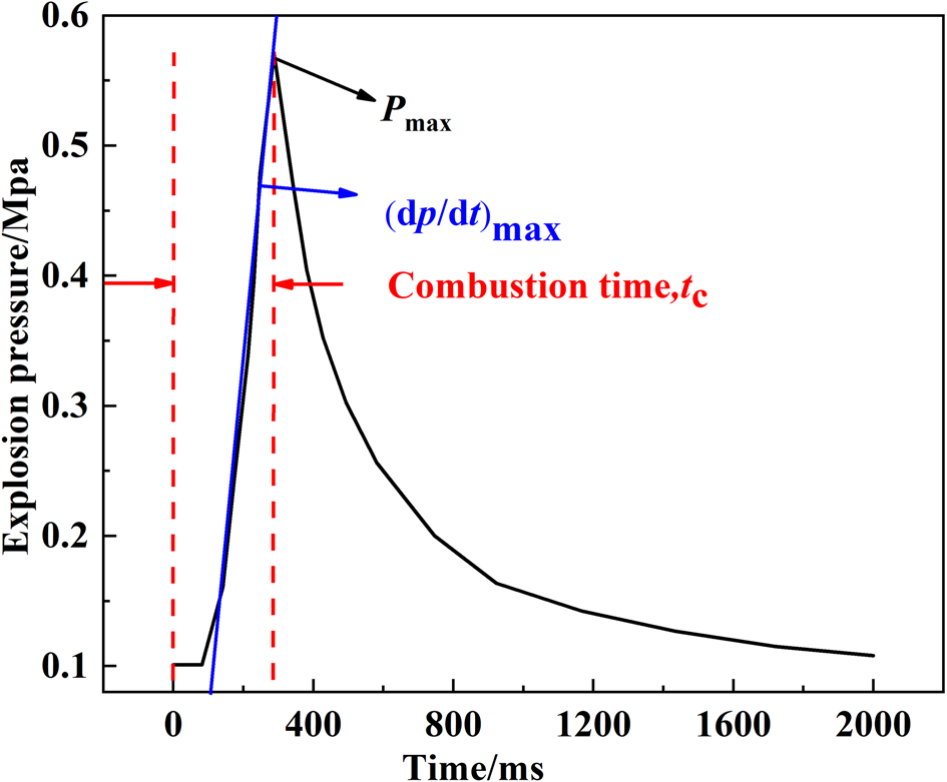
Typical pressure trajectory during an explosion.

Figure 2 shows the pressure change in the container during the explosion: after ignition, the pressure quickly reaches its maximum value. When there is no fuel left in the container, the pressure gradually drops. Figure 2 also illustrates the definition for the maximum explosion pressure *P*_max_, maximum pressure rise rate (d*P*/d*t*) max, and combustion duration *T*_c_^28,29^.

### Simulation details

#### Chemical reaction mechanism simulation

The chemical reaction mechanism simulation was calculated using the GRI-Mech3.0 reaction mechanism, and the calculation used a closed homogeneous 0-D reaction model^30^. The reactor was adiabatic. During the reaction, there was no mass or energy exchange with the surrounding environment. According to the minimum ignition temperature of methane and the ignition equipment, the initial temperature was set to 1300 K. Moreover, a small part of the mixed gas was heated to burn by setting a high-temperature heat source, with the flame rapidly spreading to the surroundings to achieve ignition. Table 1 shows the simulation parameters.

**Table 1:** Initial parameters.

Acetylene/vol.%	Methane/vol.%	Equivalent ratio	Temperature/K	Pressure/MPa	Time/s
0%	100%	0.8, 1, 1.2, 1.4	1300	0.1	0.02
20%	80%		1300	0.1	0.02
40%	60%		1300	0.1	0.02
60%	40%		1300	0.1	0.02
80%	20%		1300	0.1	0.02
100%	0%		1300	0.1	0.02

#### Mechanism verification

**Figure 3:**
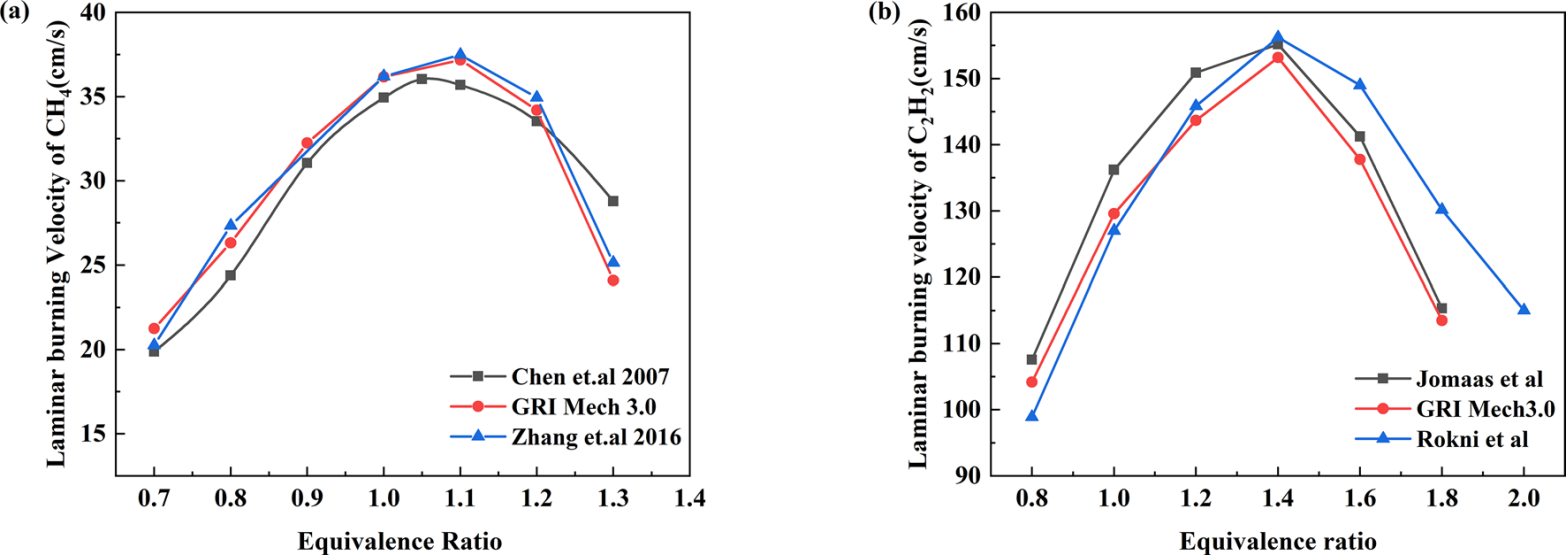
Laminar burning velocity verification [(**a**) methane, (**b**) acetylene].

At present, scholars have established many methane combustion reaction models, such as GRI3.0, DRM, USC2.0 and the Hegges mechanism. The reliability of the GRI-Mech3.0 model for the description of the chemical kinetic mechanism for methane, ethane, hydrogen and carbon monoxide gas has been verified by some scholars^31^. Therefore, this paper chose the GRI-Mech3.0 mechanism for simulation of the chemical kinetics, but this mechanism is less commonly used in acetylene, and the laminar flame velocity is a key parameter for verifying the accuracy of the chemical reaction mechanism^32^. To verify the rationality of the GRI Mech3.0 mechanism, the calculated laminar flame velocities for methane and acetylene were compared with the experimental results^33–36^. Figure 3 shows that the calculated values are consistent with the experimental results, and the components contained in the acetylene combustion process can be found in the GRI Mech3.0 mechanism. At the same time, the GRI Mech3.0 model has been applied to the combustion of propyne, indicating that this mechanism can be used to describe the mixed combustion of acetylene and methane to a certain extent^37,38^. However, as shown in Fig. 3b, due to the inconsistency of the equipment, some deviations appeared in the rich combustion state, and the experimental study mainly investigated the effect of acetylene on methane combustion, so the experiment was not designed to operate at the combustion limit of acetylene.

## Results and discussion

### Explosion limit and explosion risk

The explosion limit for combustible gas is an essential indicator for studying the hazards of flammable gas. A mixture of multiple combustible gases inevitably affects the explosion limit of methane. In most cases, the explosion limit of methane is not a fixed value. It is related to the initial temperature, pressure, ignition energy, and container size^39^. In this paper, the LEL for methane was measured to be 5.05%, and the upper explosion limit (UEL) was determined to be 14.95%, which is similar to the experimental results given in Table 2. This effect is a consequence of the environmental conditions such as relative humidity and room temperature.

**Table 2:** Explosion limit for methane in air at different initial conditions.

Vessel	Temperature/K	Pressure/MPa	Explosion limit/vol.%	References
3.4 L glass tube	298	0.1	5.15–16.15	Wang et al.^40^
20 L spherical	298	0.1	5–15	Zhang and Ng^25^
12 L spherical	308	0.1	4.9–15.8	Kondo et al.^41^
40 L cylindrical	293	0.1	4.65–15.5	Gieras et al.^42^

**Figure 4:**
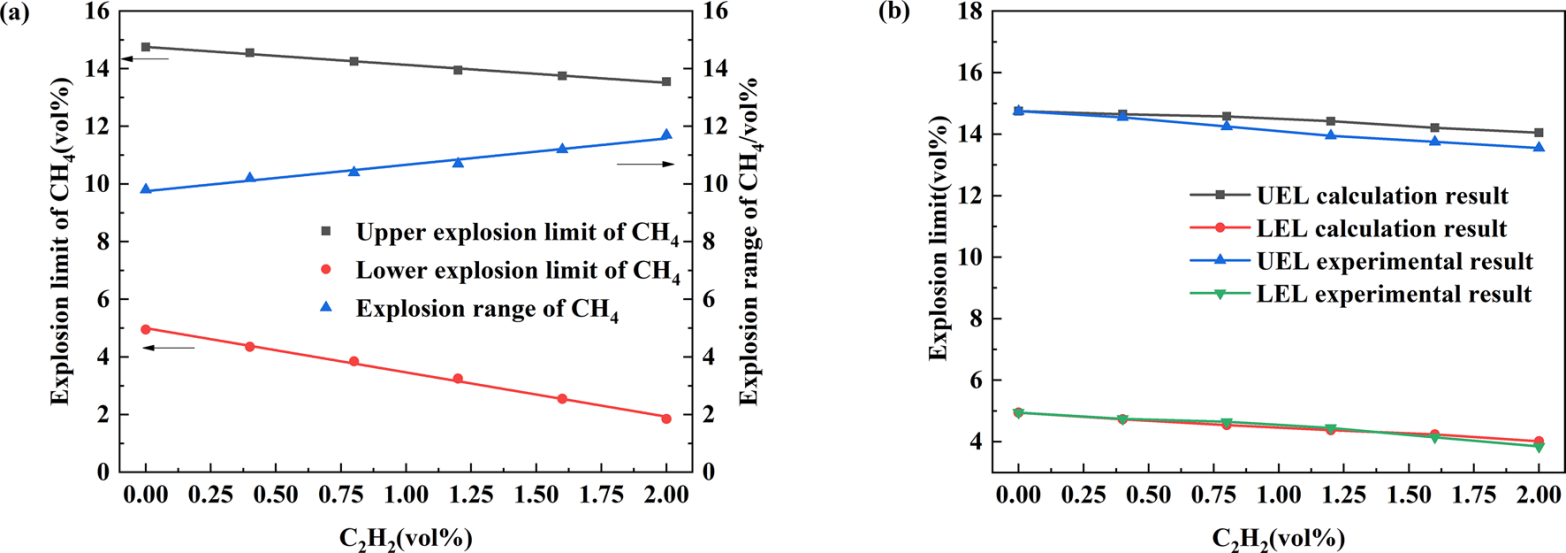
Explosion limit for methane in air [(**a**) Methane explosion limit change, (**b**) comparison of calculated and experimental values].

Figure 4a shows the change in the explosion limit of methane. This figure shows that as the acetylene concentration gradually increases, the UEL and LEL for methane have a downward trend. When the acetylene concentration increases to 2%, the LEL for methane is 1.9%, which decreases by 62.6%. The research in the literature reports the same volume fraction for combustible gas (C_2_H_6_, C_3_H_8_, H_2_) and influence of the LEL for methane as this article. Compared with the experimental results in this article, the LEL for methane under the influence of the four gases is H_2_ > C_2_H_6_ > C_2_H_2_ > C_3_H_8_^43,44^. This result also verified the Le Chatelier principle: for the same volume fractions, a gas with a lower LEL will result in the LEL of methane to decrease to a greater extent. The UEL of methane becomes 12.9%, a decrease of 12.2%. The lowering of the UEL is related to the concentration of oxygen. Due to the influence of the added gas, the proportion of air decreases, and the added gas will compete with methane to consume oxygen. Moreover, it has been found that the LEL of methane decreases more significantly relative to the UEL^26,40^. The explosion limit range for methane showed a linear upward trend, which was 13.3% larger than the explosion range for pure methane gas.

Theoretically, the explosion limit for a multicomponent combustible gas mixture can be estimated according to the Le Chatelier formula. According to Fig. 4b, the calculated value shows a linear relationship with the amount of acetylene added, which is compared with the experimental value. The error between the lower explosion limit and the calculated result was negligible. The experimental results obtained for the upper explosion limit and the calculated results have a significant error. Because Le Châtelier's model does not consider the interaction between combustible gases, the effect was not apparent at low concentrations, and the effect gradually appeared as the gas concentration increased. At the same time, some chemical effects also affected the change in the upper explosion limit^45^.

3 $$LEL_{{mix}} = 1/\sum\limits_{{i = 1}}^{n} {y_{i} /LEL_{i} }$$

4 $$UEL_{{mix}} = 1/\sum\limits_{{i = 1}}^{n} {y_{i} /UEL_{i} }$$

Here, *y*_*i*_ is the mole fraction of the ith component considering only the combustible species; LFL_*i*_ and UFL_*i*_ are the corresponding LFL and UFL, respectively.

**Figure 5:**
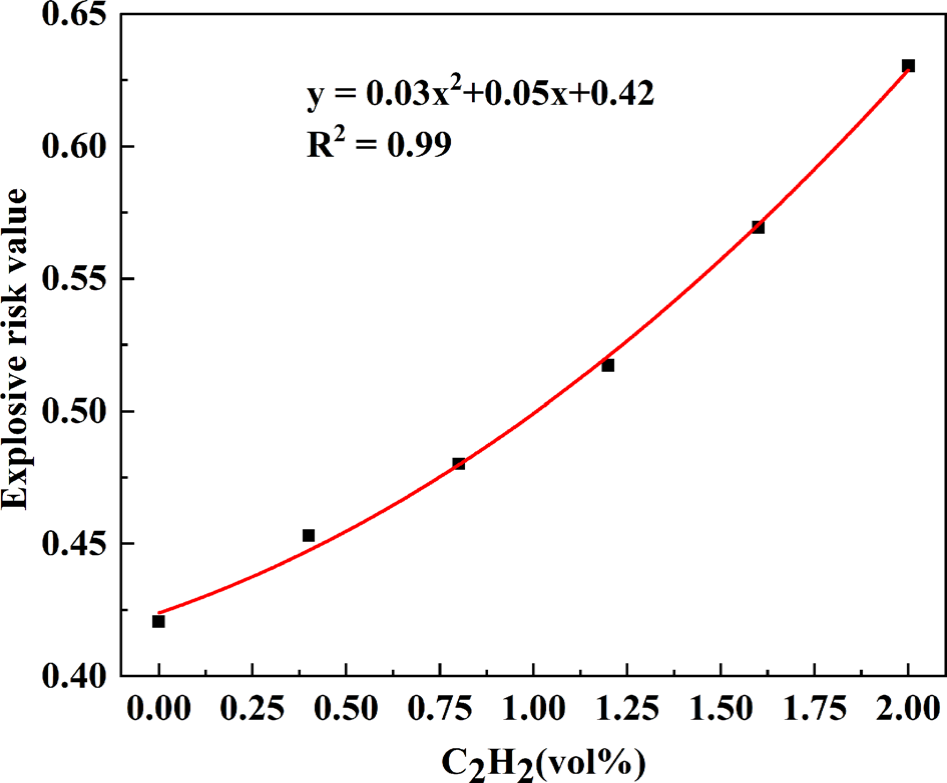
Explosive risk value of methane in air.

The relative explosion hazard F^46^ can be calculated according to the calculation formula (5), which can more intuitively indicate the change in the explosion hazard. Figure 5 shows the explosion hazard values for methane at different acetylene concentrations. As the concentration of acetylene increases, the frequency of reaction and collision of methane increases, the explosion risk for methane increases, and the rate of increase becomes increasingly faster. After fitting, it is found that the explosion hazard value has a parabolic relationship with the acetylene concentration, and the explosion hazard value for methane is increased by 47.9%.

5 $$F = {{\left[ {\left( {U \cdot L} \right)^{{0.5}} - L} \right]} \mathord{\left/ {\vphantom {{\left[ {\left( {U \cdot L} \right)^{{0.5}} - L} \right]} {\left( {U \cdot L} \right)^{{0.5}} }}} \right. \kern-\nulldelimiterspace} {\left( {U \cdot L} \right)^{{0.5}} }}$$

where *U* is the upper explosion limit for methane, %; and *L* is the lower explosion limit for methane, %.

According to the experimental results, other combustible gases will greatly reduce the LEL for methane. In most industrial environments, the concentration of methane alarm points is set below 5%. Therefore, when encountering similar situations, the monitoring and alarm threshold must be lowered to address potential explosion hazards.

### Explosion pressure parameters

**Figure 6:**
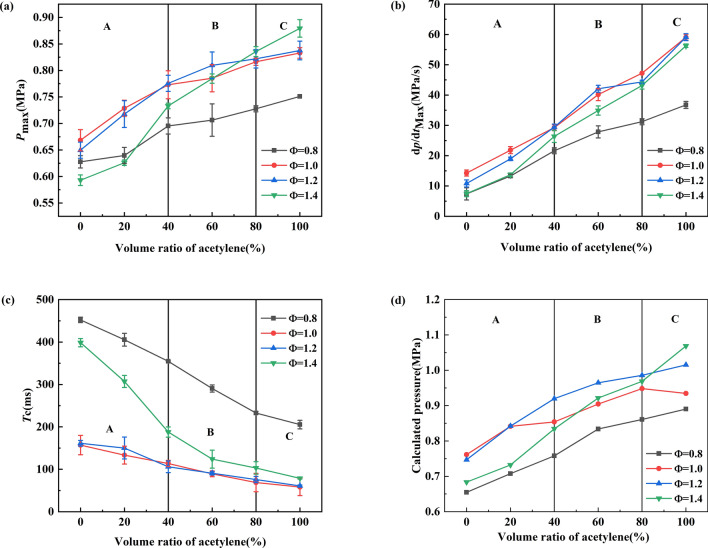
Explosion parameters for a methane–acetylene–air mixture [(**a**) *P*_max_, (**b**) (d*P*/d*t*)_max_, (**c**) *T*_c_, and (**d**) calculated pressure].

The explosion pressure parameter is an important index for studying the explosion characteristics of a gas. Figure 6 shows the changing trends for three parameters at different equivalent ratios and various volumes of acetylene–methane mixtures. For pure methane, the maximum value of *P*_max_ and (d*P*/d*t*)_max_ is approximately Φ = 1.0, and the maximum value for pure acetylene appears at Φ = 1.4. For pure methane premixed gas, the minimum combustion time *T*_c_ appears at approximately Φ = 1.0, and the minimum combustion time *T*_c_ for pure acetylene appears at approximately Φ = 1.2. The reason for this phenomenon is mainly related to the chemical properties of methane and acetylene. The combustion heat for acetylene (1368 kJ/mol) is greater than that for methane (896 kJ/mol). The *P*_max_ of pure acetylene reaches its maximum value at Φ = 1.8^33^, and *P*_max_ reaches its maximum value at approximately Φ = 1.02, which is consistent with this paper's experimental results^47^. In addition, as shown in Fig. 6d, the adiabatic pressure of the methane-acetylene mixture calculated with the GRI Mech 3.0 model shows a similar trend to that observed in the experimental results. However, the two sets of data have certain deviations in size. This difference is attributed to the heat loss in the experiment, which results in the experimental value being lower than the ideal value^48^.

An analysis of the changes in the pressure parameters shows that as the volume fraction of acetylene increases, the values of *P*_max_ and (*dP/dt*)_max_ gradually increase. The changing trend for the pressure parameters of *P*_max_ and (d*P/*d*t*)_max_ relative to the volume ratio of acetylene can be divided into three stages: (1) *P*_max_ and (d*P/*d*t*)_max_ increase slowly when the volume ratio of acetylene is less than 40%. (2) When the volume ratio of acetylene is 40–80%, *P*_max_ and (*dP/dt*)_max_ increase sharply. (3) When the volume ratio of acetylene exceeds 80%, the change in *P*_max_ slows down, while (*dP/dt*)_max_ continues to increase significantly. The increase in the volume ratio for acetylene also shortens the explosion time. The explosion time decreases exceptionally rapidly in the initial stage, and then the rate of decrease gradually slows down.

Moreover, this combustion phenomenon has also been observed in previous studies by other scholars. It is not only affected by explosion pressure parameters, but also ignition characteristics and laminar combustion speed will affect this combustion behavior, but the volume ratio of the mixed fuel will be different. For example, Qi et al.^49^ and Zhang et al.^36^ divided the combustion behaviour for premixed methane-hydrogen into three modes. Wang et al.^40^ and Luo et al.^48^ also found similar laws in the combustion of a mixed gas of methane, ethylene, and ethane. Also found similar laws for the combustion of a mixed gas of methane, ethylene, and ethane. From the above analysis, in terms of the volume ratio of acetylene, this article divided the combustion behaviour into three combustion phases: (A) For the combustion phase, where the volume ratio of acetylene is less than 40%, the combustion phase is dominated by methane; (B) the transition phase occurs from 40 to 80% of the volume ratio of acetylene; (C) for the stage where the volume fraction of acetylene exceeds 80%, acetylene dominates the combustion of the mixed gas.

According to this law, we can apply the combustion behaviour to a broader range of binary mixtures, which will more accurately predict the combustion and explosion characteristics of the corresponding proportion of the mixture and provide a theoretical basis for preventing multigas explosions.

### Laminar burning velocity

The laminar burning velocity (*S*_*L*_) refers to the flat, unstretched flame surface velocity relative to the unburned premixed gas under adiabatic conditions. It is one of the crucial parameters reflecting the combustion characteristics of combustible gas and is essential for analysing and calculating explosion disasters. The laminar burning velocity can be obtained by analysing and calculating the trajectory of the spherical flame expansion. It can also be calculated according to the explosion pressure–time curve^50^. Formula (4) shows the mathematical expression for the model. The literature shows that this model's laminar burning velocity is not much different from the results obtained by other methods^35,51–53^. *S*_*L*_ was calculated when the flame radius is greater than 6 mm to avoid the effects related to spark ignition, so the result can be considered an ideal spherical flame propagating outwards^25,54^.

6 $$S_{L} = \frac{1}{{P_{{\max }} - P_{0} }} \cdot \frac{1}{3}\left( {\frac{{4\pi }}{{3V}}} \right)^{{ - 1/3}} \cdot \left( {\frac{{P_{0} }}{P}} \right)^{{1/\gamma }} \cdot \left[ {1 - \left( {\frac{{P_{0} }}{P}} \right)^{{1/\gamma }} \cdot \left( {\frac{{P_{{\max }} - P}}{{P_{{\max }} - P_{0} }}} \right)} \right] \cdot \frac{{dP}}{{dt}}$$

where *P*_max_, *P*_0_, and *P* are the maximum explosion pressure, initial pressure, and actual pressure, respectively; *V* is the explosion chamber volume; *γ* is the adiabatic index of the unburned gas; and d*P*/d*t* is the rate of pressure increase.

**Figure 7:**
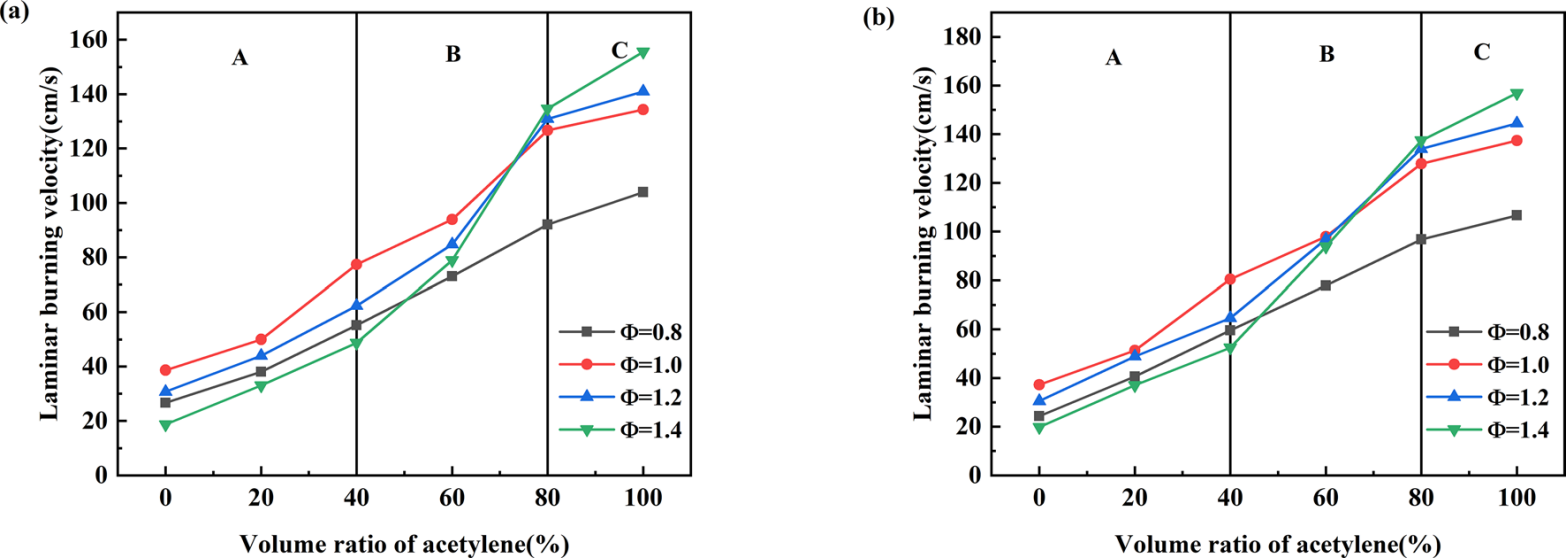
*S*_*L*_ value of mixed gas [(**a**) *S*_*L*_ from spherical flame method, (**b**) *S*_*L*_ from GRI Mech 3.0].

**Figure 8:**
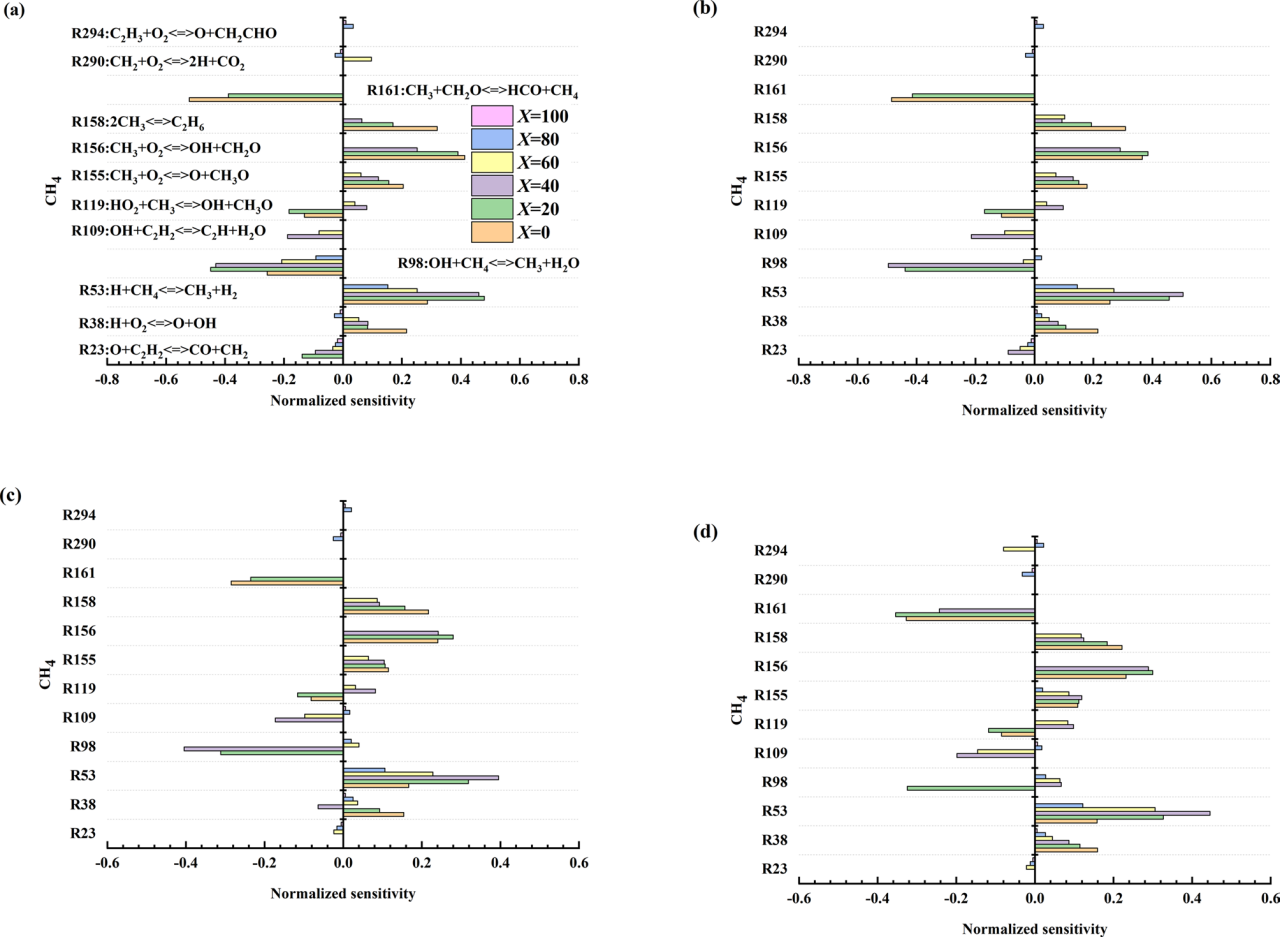
Normalized methane sensitivity coefficients for four equivalent ratios [(**a**) Φ = 0.8, (**b**) Φ = 1.0, (**c**) Φ = 1.2, (**d**) Φ = 1.4] during explosion.

This paper used this calculation method to calculate the laminar burning velocity of the methane-acetylene mixture and compared it with the calculation result obtained from GRI Mech3.0. Figure 7 shows the laminar burning velocity of the methane-acetylene-air mixture equivalent to 0.8–1.4 determined using the two calculation methods. It is derived from Fig. 8 that the laminar burning velocity of the mixture increases as the volume percentage of acetylene increases. In the combustion phase dominated by methane, the maximum and minimum values for *S*_*L*_ correspond to equivalent ratios of 1.0 and 1.4, respectively; during the transition period and the combustion phase dominated by acetylene, the *S*_L_ value with an equivalent ratio of 1.2 gradually exceeds the equivalent ratio *S*_*L*_ value of 1. The minimum value of *S*_*L*_ also changes from an equivalent ratio of 1.4 to 0.8. Comparing Fig. 7a with Fig. 7b, it is found that the *S*_*L*_ value of Fig. 7a is greater than Fig. 7b. Two factors cause this phenomenon. One is that the GRI Mech 3.0 model does not heat loss. The other is that spherical flame method does not consider the Markstein length correction and the direction of flame propagation.

### Sensitivity analysis

Sensitivity analysis mainly studies the changes in the reaction of the main elements in the gaseous mixture combustion process. This paper analyses the sensitivity of methane in detail to further study the reaction mechanism of a methane-acetylene-air mixed explosion. Since the magnitude of the sensitivity of each reaction is very different, it cannot be fully expressed in the same figure, so the sensitivity value is normalized, and the normalized sensitivity value is obtained, using the formula:

7 $$x^{\prime} = \frac{{x - {\text{mean}}(x)}}{{\max (x) - \min (x)}}$$

Figure 8 shows that under the conditions of four equivalent ratios, the main reactions that promote methane consumption are R38, R53, R156, R155, and R158, and that the reactions that promote methane production are R23 and R161. Under different equivalent conditions, the key elements that affect the methane reaction generally remain unchanged. Some reactions increase with the equivalent ratio. In some reactions, with a change in the equivalence ratio, the sensitivity is reversed, for example, for reactions R98 and R38.

The study also found that the sensitivity coefficient for most reactions decreases with increasing acetylene volume fraction. In addition, the volume fraction of acetylene has an undeniable impact on the sensitivity of methane. As the volume fraction increases, the sensitivity of some reactions reverses. For example, when the volume fraction of acetylene is 40%, the sensitivity of R119 is changed. Moreover, part of the reaction is replaced when the volume fraction is 10%, 40%, and 80%. This article believes that the above changes should have a specific relationship with the combustion stage. This change can provide theoretical support for studying the combustion stage of binary gases.

### Effect of acetylene addition on rate of production

Current research for the mechanism of methane explosion mainly focuses on the formation of intermediate products and the reaction pathways among O, H, and OH radicals. In the methane explosion process, especially in the induction period of the methane explosion, free radical reactions dominate the chain reaction. The concentration of free radicals also has a significant impact on methane explosions. In the study of Luo et al.^55^ it was found that with the pyrolysis of the fuel, the early chain reaction will generate a large number of free radicals at high temperatures. The concentration of free radicals shows a steep peak; then, some free radicals are consumed in the chain reaction, and the concentration of free radicals is reduced to an absolute value. This article mainly studies the effect of acetylene on free radical productivity.

**Figure 9:**
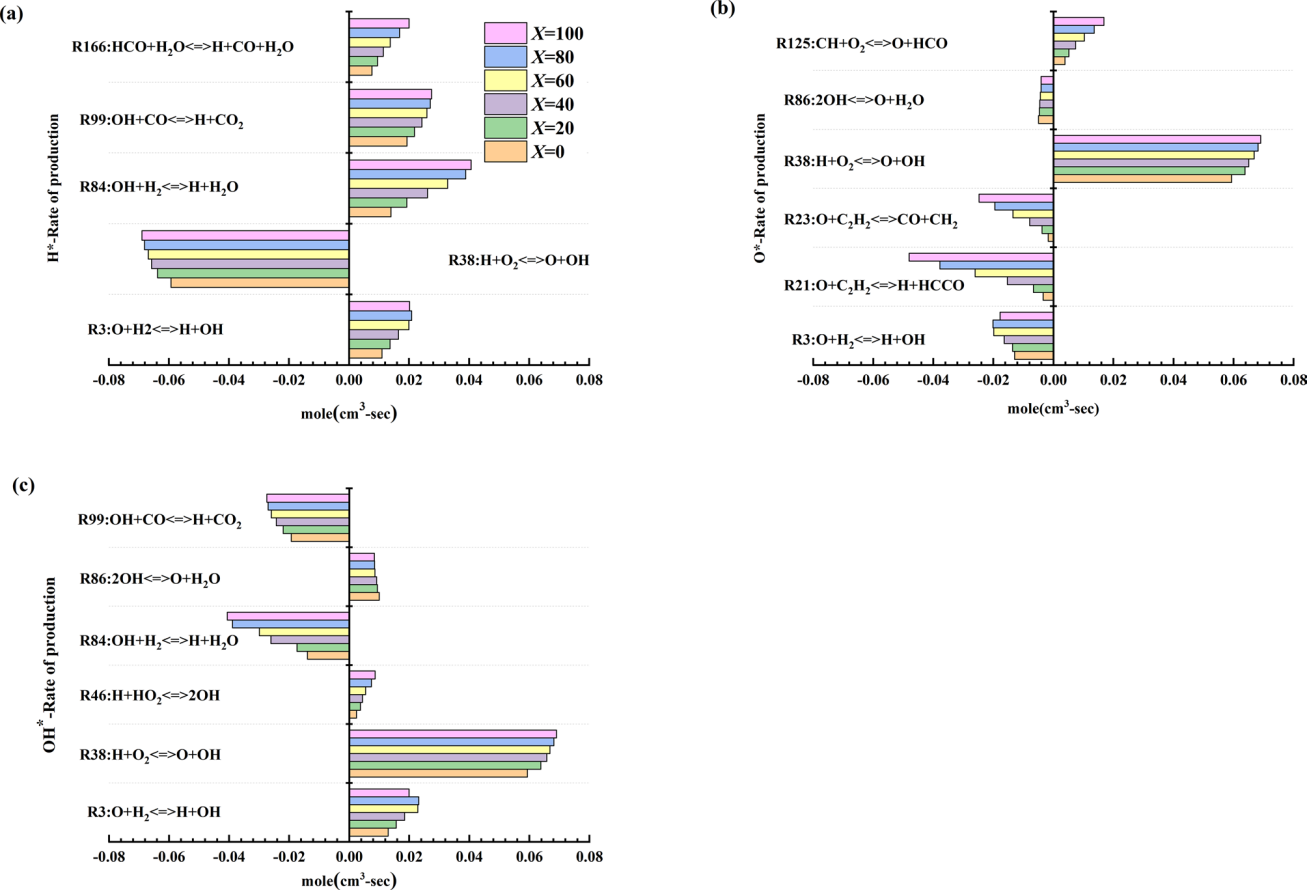
Rates of production and consumption of free radicals [(**a**) H*, (**b**) O*, (**c**) OH*].

An analysis of the changes in free radical reactions with different equivalent ratios found that the main reactions affecting free radical production and consumption rates (H*, O*, and OH*) are the same. Therefore, this article takes an equivalent ratio of 1 as an example and selects several iconic reactions to analyse the free radical generation rate changes. Figure 9 shows the changes in the production and consumption rates for H*, O*, and OH* for an equivalent ratio of 1. Figure 9 shows that the main element reactions that affect the rate of free radical generation and consumption during the explosion reaction of the methane-acetylene mixture are R3, R21, R23, R38, R46, R58, R84, R86, R99, R125, and R166. When the rate value is positive, the reaction increases the rate of production of free radicals; when the rate value is negative, the reaction promotes free radical consumption. Among all the reactions, the most critical reaction that promotes H* radical consumption is R38, which is also the most critical reaction to generate O* and OH*.

In addition, free radical productivity is also affected by the three combustion stages. As the proportion of acetylene increases, the production rate of the reaction increases and replaces part of the main reaction. When methane dominates the combustion, reactions R10 (O + CH_3_ <=> H + CH_2_O) and R167 (HCO <=> H + CO) occupy part of the main position; in the transitional period, reactions R7(O + CH_2_ <=> H + HCO) and R126 (CH + H_2_ <=> H + CH_2_)replace some reactions; when acetylene dominates the combustion process, reactions R28 (O + HCCO <=> H + 2CO), R109, R144 (CH_2_ + O_2_ <=> H + OH + CO), and R290 rise to the main position in the reaction. This change verifies the division of the explosion phase of the methane-acetylene-air mixture described in the above text.

Overall, when the equivalent ratio is the same, as the volume fraction of acetylene increases, the rate of free radical production and consumption gradually increases. This result leads to a gradual increase in the chemical reaction rate for the mixed gas and shortens the explosion time for the mixed gas. Simultaneously, the increase in maximum explosion pressure and laminar flame propagation speed also reflects the increase in productivity and consumption rate.

Therefore, according to the change in methane sensitivity and the change trend for free radicals, some measures can be taken at the molecular level to prevent and control explosion accidents at the initial stage of a gas explosion, thereby effectively reducing the explosion risk.

## Conclusion

This paper studied the explosion characteristics of methane-acetylene-air mixtures. All experiments were carried out in the same environment. The experimental results included the mixed gas explosion limit, explosion pressure parameters, laminar burning velocity. And through sensitivity analysis, the main element reactions that affects the gas reaction were obtained. The main conclusions are as follows:

1. The increase in the acetylene volume fraction leads to a gradual decrease in the explosion limit for methane, and the lower explosion limit is more obvious to the upper limit. The explosion hazard coefficient for the mixed gas shows a parabolic upward trend.
2. Under the four equivalent ratios, as the volume ratio of acetylene increases, the maximum explosion pressure and the maximum explosion rise rate continuously increase, and the explosion reaction time decreases. Analysis of the explosion pressure parameters enabled determination of the three combustion stages for the methane-acetylene-air mixture.
3. As the volume ratio of acetylene increases, the mixed gas laminar burning velocity increases. When the equivalent ratio of pure methane is 1.0, the laminar burning velocity is the largest. The laminar burning velocity of pure acetylene is largest when the equivalent ratio is 1.2.
4. Under different equivalence ratio conditions, the main basic reactions that affect methane sensitivity and the rate of free radical generation are the same. The sensitivity of some reactions is reversed. Moreover, the most important reactions that affect the sensitivity of methane were identified; they help the explosion. Under the same equivalent ratio, with an increase in the volume fraction of acetylene, the generation rate and consumption rate of free radicals (H*, O* and OH*) increase at the same time, which increases the reaction rate, and a part of the main reaction is replaced.

Although the current research reached the above conclusions, there are still many shortcomings. In the future, we plan to conduct in-depth research into the propagation law for methane-acetylene-air mixtures in pipe networks and the explosion mechanism.
